# Peri-Operative Outcomes Associated With the Placement of Implantable Cardioverter-Defibrillators in Patients With Sarcoidosis: A Nationwide Database Analysis

**DOI:** 10.7759/cureus.55589

**Published:** 2024-03-05

**Authors:** Shreyas Singireddy, Samuel Edusa

**Affiliations:** 1 Internal Medicine, Piedmont Athens Regional, Athens, USA

**Keywords:** national inpatient sample (nis), post-op complication, cardiac arrhythmias (ca), extrapulmonary sarcoid, implantable cardioverter-defibrillator (icd)

## Abstract

Rationale

Sarcoidosis with cardiac involvement can be associated with serious life-threatening arrhythmias and an increased risk of sudden cardiac death. Implantable cardiac defibrillators (ICDs) have been used for primary and secondary prevention of sudden death in patients with cardiac sarcoidosis (CS). Post-ICD placement complications have been shown to be higher in patients with CS. However, data comparing postoperative ICD complications among sarcoidosis patients with the general population is limited. Here, we evaluated the association of postoperative complications with implantable cardioverter-defibrillators in sarcoidosis.

Methods

Using the NIS database, we identified cases of adults aged ≥ 18 years undergoing surgical placement of implantable cardioverter-defibrillators between 2010 and 2019. Using ICD-9 and ICD-10 codes, we identified patients with sarcoidosis. In all statistical analyses, we applied weights provided by HCUP to produce results representative of national estimates. We compared categorical and continuous covariates in the baseline characteristics using the chi-square test and analysis of variance, respectively. We employed multivariable logistic and linear regression to compare binomial and continuous outcomes to assess differences in mortality rates.

Results

We analyzed 114073 patients during the study period. Of these, 1012 (0.9%) had sarcoidosis and were found to be significantly younger and female compared to patients without sarcoidosis (56.4 ±11.5 years vs. 65.6 ± 13.9 years, p <0.001) and (39.4% vs. 28.3%, p<0.001) respectively. Further, patients with sarcoidosis were more likely to be African American (45% vs. 16.3%), have private insurance (45.4% vs. 23.8%), and less likely to have Medicare (34.9% vs. 60.9%). Overall, post-ICD placement complications such as lead complications (4.2% vs. 6.9%, p = 0.03), post-procedure hemorrhage (4.1% vs. 5.5%, p=0.048), and requirement for transfusion (2.3% vs. 4.4%) were less likely in patients with sarcoidosis. Regarding post-ICD placement inpatient mortality, sarcoidosis was not associated with any difference (OR: 0.71, 95% CI 0.18-2.88 p=0.634).

Conclusions

Placement of implantable cardioverter-defibrillators in patients with sarcoidosis is a safe procedure and is associated with significantly lower rates of lead complications, post-procedure hemorrhage, and requirement for transfusion. This is of great importance as it is known that patients with underlying sarcoidosis are predisposed to developing more cardiac complications.

## Introduction

Sarcoidosis is a multi-system inflammatory granulomatous disease of unknown etiology [1]. An estimated 5% of patients with sarcoidosis develop cardiac involvement with associated complications, including life-threatening arrhythmias, heart block, cardiomyopathy, heart failure, and sudden death [2]. The most recent guidelines give a Class 1 recommendation for implantable cardiac defibrillator placement in patients with an ejection fraction less than 35% based on a 25-year study done in Finland in patients with underlying cardiac sarcoidosis and heart failure as marked LV dysfunction correlated to higher adverse outcomes [3]. In addition, patients can experience ventricular arrhythmias and sudden cardiac death even if the ejection fraction is normal, which further necessitates the need for ICD placement [4]. The high rate of high-risk cardiac outcomes in this population highlights the need to evaluate the overall safety and assess the rate of complications of ICD placement in this group of patients. This study aims to compare complication rates between sarcoidosis patients and those without the disease undergoing ICD placement.

## Materials and methods

Data source

For the current project, the authors used the National Inpatient Sample (NIS) database. The Agency for Healthcare Research and Quality (AHRQ) maintains the NIS, a free, all-payer inpatient database (AHRQ). The NIS represents a stratified sample of 20% of community hospitals in the United States and is made up of discharge-level data from about 8 million hospitalizations each year. As a result of the NIS's sampling approach, it is possible to apply weighting variables to the calculation of national estimates, which have been verified against other US hospital registries. Every hospitalization in the database has clinical and resource-use data, such as the patient's age, sex, race, insurance status, primary and secondary procedures, hospitalization outcome, overall cost, and duration of stay, among other things. The International Classification of Diseases, Ninth Revision, Clinical Modification (ICD-9-CM) and Tenth Revision (ICD-10-DR) are used to record patient diagnoses. Given that NIS is a deidentified dataset, we did not seek nor require IRB approval.

Cohort selection

Using the ICD-9 (379.4, 379.5, 379.6, 00.51) and ICD-10 (02H40KZ, 02H43KZ, 02H44KZ, 02HK0KZ, 02HK3KZ, 02HK4KZ, 02HL0KZ, 02HN0KZ) procedures codes, we identified all adult (≥18) patients undergoing surgical placement of implantable cardioverter-defibrillators between 2010 and 2019. Next, we identified patients with Sarcoidosis (ICD-9 code: 135; ICD-10 diagnosis codes: D860, D861, D862, D863, D868, D8681, D8682, D8683, D8684, D8685, D8686, D8687, D8689, D869). Comorbidities that are historically associated with worse procedural outcomes were selected. Mechanical complications, hematoma formation, lead or pocket revision, injury to blood vessels, pneumothorax, pericardial complications, bleeding necessitating transfusion, ICD infection, in-hospital mortality, and length of stay were all common in-hospital complications of ICD placement that were reported. ICD-9 and ICD-10 diagnosis and procedure codes that correlate to these problems allowed for their identification.

National estimates

Using a tried-and-true sample weight method, we calculated the prevalence of sarcoidosis (both overall and with cardiac involvement) at the population level. To help analysts and investigators examine national patterns over time, the NIS provides a mechanism to deduce national-level estimates from these data. This process is made possible by a variable called TRENDWT (trend weight) prior to 2012 and DISCWT (discharge weight) following 2012. Using this method, national estimates (weighted discharges) are created from the number of incidents recorded in the NIS (unweighted).

Outcomes of interest

The occurrence of complications and in-hospital mortality between the two groups was evaluated. We also performed multivariable analyses to evaluate the effect of sarcoidosis on in-hospital mortality.

Statistical analysis

In all statistical analyses, we applied weights provided by HCUP to produce results representative of national estimates. We compared categorical and continuous covariates in the baseline characteristics using the chi-square test and analysis of variance, respectively. To evaluate the impact of sarcoidosis on mortality, multivariable logistic regression was employed. We decided to incorporate all clinically important variables in our multivariable model in accordance with modern statistical approaches rather than choosing variables based on univariate analysis.

## Results

A total of 114073 procedures between 2010 and 2019 were identified. Of these, 1012 (0.9%) were found to have sarcoidosis, while the remaining 113061 procedures (91.1%) did not have underlying sarcoidosis. 

Demographic characteristics

Patients with sarcoidosis were found to be significantly younger compared to patients without sarcoidosis (56.4 ± 11.5 years vs 65.6 ± 13.97, p<0.001). Patients with sarcoidosis were more likely to be females (39.4% vs 28.3%, p<0.001). Patients with sarcoidosis were more likely to be African Americans (45% vs 16.3%) and less likely to be White (47.5% vs 70.1%) (overall p<0.001). Patients with sarcoidosis were more likely to have private insurance (45.4% vs 23.8%) and less likely to have Medicare (34.9% vs 60.9%) (overall p<0.001). Patients with sarcoidosis were more likely to have an emergency admission (77.5% vs 70%, p<0.001) (Table 1).

**Table 1:** Underlying demographic characteristics of patients with and without sarcoidosis

	No Sarcoidosis (N=113061)	Sarcoidosis (N=1012)	Total (N=114073)	p-value
Age				< 0.001
Mean (SD)	65.573 (13.972)	56.441 (11.537)	65.492 (13.978)	
Range	0 - 98	20 - 88	0 - 98	
Female	32008 (28.3%)	399 (39.4%)	32407 (28.4%)	< 0.001
Race				< 0.001
White	73879 (70.1%)	454 (47.5%)	74333 (69.9%)	
African American	17155 (16.3%)	430 (45.0%)	17585 (16.5%)	
Hispanic	8534 (8.1%)	27 (2.8%)	8561 (8.1%)	
Asian	2051 (1.9%)	13 (1.4%)	2064 (1.9%)	
Native American	526 (0.5%)	3 (0.3%)	529 (0.5%)	
Other	3199 (3.0%)	29 (3.0%)	3228 (3.0%)	
Insurance				< 0.001
Medicare	68722 (60.9%)	353 (34.9%)	69075 (60.6%)	
Medicaid	10887 (9.6%)	130 (12.9%)	11017 (9.7%)	
Private Insurance	26861 (23.8%)	459 (45.4%)	27320 (24.0%)	
Self-Pay	3274 (2.9%)	31 (3.1%)	3305 (2.9%)	
No Charge	373 (0.3%)	3 (0.3%)	376 (0.3%)	
Other	2772 (2.5%)	35 (3.5%)	2807 (2.5%)	
Admission				< 0.001
Emergency Admission	78803 (70.0%)	782 (77.5%)	79585 (70.1%)	
Elective Admission	33719 (30.0%)	227 (22.5%)	33946 (29.9%)	

Comorbidities

Patients with sarcoidosis were more likely to have congestive heart failure (CHF) (79.2% vs 42.9%, p<0.001) and ventricular tachycardia (43.6% vs 38.3%, p<0.001), and less likely to have myocardial infarction (MI) (10.3% vs 25.9%), peripheral vascular disease (PVD) (1.6% vs 6.6%, p<0.001), stroke (3.1% vs 6.1%, p<0.001), dementia (0.3% vs 0.9%, p<0.001), diabetes with complication (12.7% vs 22%, p<0.001), renal disease (18.8% vs 23.9%, p<0.001), atrial fibrillation (19.5% vs 31.3%, p<0.001), ventricular fibrillation (8% vs 12.2%, p<0.001), and cardiac arrest (5.5% vs 9.1%, p<0.001) (Table 2).

**Table 2:** Pre-existing comorbidities among patients with and without sarcoidosis Myocardial infarction (MI), peripheral vascular disease (PVD), congestive heart failure (CHF)

	No Sarcoidosis (N=113061)	Sarcoidosis (N=1012)	Total (N=114073)	p value
MI	29302 (25.9%)	104 (10.3%)	29406 (25.8%)	< 0.001
CHF	34067 (42.9%)	446 (79.2%)	34513 (43.2%)	< 0.001
PVD	5218 (6.6%)	9 (1.6%)	5227 (6.5%)	< 0.001
Stroke	6939 (6.1%)	31 (3.1%)	6970 (6.1%)	< 0.001
Dementia	1042 (0.9%)	3 (0.3%)	1045 (0.9%)	0.038
Chronic Pulmonary Disease	24927 (22.0%)	210 (20.8%)	25137 (22.0%)	0.322
Connective Tissue Disease/ Rheumatic Disease	2151 (1.9%)	16 (1.6%)	2167 (1.9%)	0.456
Peptic Ulcer Disease	232 (0.2%)	2 (0.2%)	234 (0.2%)	0.958
Diabetes w Complications	7510 (22.3%)	57 (12.7%)	7567 (22.2%)	< 0.001
Renal Disease	18961 (23.9%)	106 (18.8%)	19067 (23.8%)	0.005
Cancer	1183 (1.0%)	11 (1.1%)	1194 (1.0%)	0.899
Mild Liver Disease	2298 (2.0%)	25 (2.5%)	2323 (2.0%)	0.326
Moderate Severe Liver Dx	264 (0.3%)	5 (0.9%)	269 (0.3%)	0.023
Metastatic Carcinoma	131 (0.1%)	2 (0.2%)	133 (0.1%)	0.448
AIDS/HIV	127 (0.2%)	0 (0.0%)	127 (0.2%)	0.342
Atrial Fibrillation	35421 (31.3%)	197 (19.5%)	35618 (31.2%)	< 0.001
Ventricular Tachycardia	43298 (38.3%)	441 (43.6%)	43739 (38.3%)	< 0.001
Ventricular Fibrillation	13787 (12.2%)	81 (8.0%)	13868 (12.2%)	< 0.001
Cardiac Arrest	10330 (9.1%)	56 (5.5%)	10386 (9.1%)	< 0.001

Peri-operative complications

Most complications were found to have a similar rate of occurrence between the two groups. Patients with sarcoidosis were less likely to have lead complications (4.2% vs 6.9%, p=0.026), post-procedural hemorrhage (4.1% vs 5.5%, p=0.048), and requirement for transfusion (2.3% vs 4.4%, p=0.001). Upon multivariable analysis, sarcoidosis was not found to have any effect on the odds of mortality (OR 0.71, p=0.634) (Table 3).

**Table 3:** Peri-operative complications during ICD placement procedure between patients with and without sarcoidosis

	No Sarcoidosis (N=113061)	Sarcoidosis (N=1012)	Total (N=114073)	p-value
Mechanical Complication	5668 (5.0%)	45 (4.4%)	5713 (5.0%)	0.411
Lead Complication	2328 (6.9%)	19 (4.2%)	2347 (6.9%)	0.026
Lead Revision	5445 (4.8%)	51 (5.0%)	5496 (4.8%)	0.741
Pneumothorax	1431 (1.3%)	12 (1.2%)	1443 (1.3%)	0.821
Hemopericardium	59 (0.1%)	0 (0.0%)	59 (0.1%)	0.467
Pericardial Tamponade	240 (0.2%)	1 (0.1%)	241 (0.2%)	0.434
Unspecified Pericardial Tamponade	87 (0.3%)	0 (0.0%)	87 (0.3%)	0.281
Pericardiocentesis	243 (0.2%)	0 (0.0%)	243 (0.2%)	0.14
Post Procedural Hemorrhage	6183 (5.5%)	41 (4.1%)	6224 (5.5%)	0.048
Transfusion	4941 (4.4%)	23 (2.3%)	4964 (4.4%)	0.001
In-Hospital Mortality	861 (0.8%)	3 (0.3%)	864 (0.8%)	0.089

## Discussion

This analysis of the National Inpatient Sample (NIS) data, a stratified representative sample of 20% of community hospitals across the United States, demonstrates the safety and efficacy of implantable cardioverter-defibrillators (ICDs) in the context of patients with underlying sarcoidosis, considering their characteristic demographic and comorbidity profile. With the inclusion of a total of 114,073 discharges over a nine-year period (2010 to 2019), our study represents one of the most comprehensive research investigations to date into the safety implications of ICD implantation in sarcoidosis patients.

Previous research has suggested a demographic trend among sarcoidosis patients, who are typically younger, more likely to be female, and African American [3,5]. Our study corroborates these findings and emphasizes the need to consider these demographic factors when assessing the safety and efficacy of treatments such as ICDs. Furthermore, our study provides additional insights into the distinct comorbidity profile of these patients. Despite their relatively younger age, sarcoidosis patients have a significantly higher burden of cardiac comorbidities, such as CHF (79.2% vs 42.9%, p<0.001) and ventricular tachycardia (43.6% vs 38.3%, p<0.001). This finding is consistent with prior research demonstrating the profound effect of sarcoidosis on the cardiovascular system and its potential to lead to life-threatening complications [6].

Clinical involvement of the cardiac system has been reported in 5-10% of patients with underlying systemic sarcoidosis [7]. Furthermore, evidence of clinically undiagnosed involvement of the cardiac system was seen on autopsy reports in 25% of the patients with systemic sarcoidosis [8]. These numbers emphasize the burden of unrecognized cardiac involvement in patients with sarcoidosis. Our analysis demonstrated an overall incidence of ventricular tachycardia in 43% of the patients with underlying sarcoidosis compared to 38% in patients without, thus pointing towards the need to make a better attempt at evaluating patients with sarcoidosis.

The data from our study revealed that the complex disease profile of sarcoidosis patients, including the high burden of cardiac comorbidities, does not contribute to increased complications associated with ICD placement. In fact, some complications had a significantly lower rate in the sarcoid population, such as lead complications, post-procedure hemorrhages, and the need for blood transfusions. These findings suggest that while patients with sarcoidosis pose significant clinical challenges, the safety of ICD implantation is not compromised. Rather, this critical life-saving intervention can be successfully and safely executed even in these complex cases, providing hope and an effective treatment strategy for clinicians and patients alike. Reasons explaining the lower rates of complications could be secondary to the lower burden of pre-existing comorbidities in the cohort with sarcoidosis. These findings go hand in hand with the most recent AHA/ACC guidelines regarding ICD placement cardiac sarcoidosis with a 1B recommendation being implantation of an ICD for patients with a history of sustained ventricular tachycardia, prior history of sudden cardiac arrest, and in those whose ejection fraction is 35% or less [9]. Overall, the recommendations for ICD placement range from I to IIB based on underlying features per the 2017 AHA/ACC guidelines.

Another finding of our study that warrants attention is the higher frequency of emergency admissions among sarcoidosis patients (77.5% vs 70%, p<0.001). This may be reflective of the unpredictable and severe course of cardiac sarcoidosis, necessitating immediate medical attention and treatment. Interestingly, despite the emergent nature of these admissions and the inherent complexities of providing acute care, there was no significant association between emergency admission and ICD placement complications. This finding underlines the robustness of ICD placement as an effective life-saving intervention, even in acute care settings, and reaffirms its importance in the clinical management of sarcoidosis.

The insights derived from our study build upon and substantiate the findings of earlier smaller-scale studies, demonstrating the safety and efficacy of ICDs in preventing sudden cardiac death in patients with sarcoidosis [10,11]. By providing additional compelling evidence, our analysis contributes to the growing body of literature that supports the recommendation for considering ICDs as a viable intervention in sarcoidosis patients at risk of life-threatening arrhythmias. The significance of this recommendation is heightened in the light of increasing recognition of sarcoidosis as a cause of heart failure and sudden cardiac death, coupled with the observed rise in the use of ICDs in this patient group.

Regarding future directions, positron emission tomography could be considered in terms of prognostication as these recent studies show that the coexistence of left ventricular (LV) scar and active inflammation, results in better prediction of outcomes in comparison to LV scar alone [12,13]. In addition, evaluation of other factors such as oxidative stress and features of the right ventricle (RV) also need to be incorporated as more data is pointing towards the effect of certain RV features playing a role in the evaluation of prognosis in these groups of patients [14,15,16].

While our study contributes significantly to the understanding of ICD safety in sarcoidosis patients, it is important to note certain inherent limitations. As with any retrospective study, ours is also susceptible to potential selection bias, given that only the sickest sarcoidosis patients are likely to have undergone ICD placement. Additionally, despite the large scale and comprehensiveness of the NIS dataset, it does not provide detailed information about the specifics of sarcoidosis, such as the severity of the disease or the involvement of organs beyond the heart. The lack of these granular data may limit the depth of understanding regarding the complex interplay of these factors in the observed outcomes. Moreover, the NIS dataset primarily focuses on the inpatient experience, limiting the capacity to evaluate long-term outcomes, including morbidity and mortality post-discharge. To address these limitations and provide a more comprehensive picture, future prospective studies should focus on assessing the long-term outcomes and potential complications associated with ICDs in sarcoidosis patients. Of note, this article was posted to a pre-publication server on August 17, 2023

## Conclusions

In conclusion, the evidence presented in our study suggests that ICD placement is safe in sarcoidosis patients and is not associated with an increased risk of in-hospital morbidity or complications. This finding holds true despite the higher prevalence of cardiac comorbidities among these patients, signifying the procedure's safety even in complex clinical scenarios. Therefore, this data should encourage clinicians to consider ICD implantation as a life-saving intervention in sarcoidosis patients at risk of life-threatening arrhythmias. Looking forward, the research agenda should focus on evaluating long-term outcomes and further defining the subgroups of sarcoidosis patients who might derive the most benefit from ICD implantation, to ensure optimal patient management and care.
